# A Manual of Ophthalmoscopy

**Published:** 1902-07

**Authors:** 


A Manual of Ophthalmoscopy. For Students and General Practitioners. By J. E. Jennings, M. D. (Univ. of Penna.), Author of Color-Vision and Color-Blindness, etc.; formerly Clinical Assistant, Royal London Ophthalmic Hospital. Duodecimo, pp. 180, with 95 illustrations and one colored plate. Philadelphia: P. Blakiston's Son & Co. 1902. [Price, $1.25 net.]

A knowledge of the use of the ophthalmoscope should be as common as that of the stethoscope—and not be limited to the ophthalmologists. At the present time the use of this instrument is being taught in our medical schools, and this manual is an example of what is being done in some of the western colleges. Dr. Jennings is to be congratulated on his efforts and also that he is striving to disseminate knowledge on this subject. The text is systematically arranged and profusely illustrated with many original black-and-white drawings and an excellent colored frontispiece. W.C.K.

